# A Treatise on the Principles and Practice of Medicine

**Published:** 1909-12

**Authors:** 


**A Treatise on the Principles and Practice of Medicine.** By **Arthur R. Edwards,** M.D., Professor of the Principles and Practice of Medicine and Clinical Medicine in the Northwestern University Medical School, Chicago. Second edition. Octavo, 1246 pages, with 100 engravings and 21 full-page plates in colors and monochrome. Lea & Febiger, Philadelphia and New York, 1909. (Cloth, $5.50, net; leather, $6.50, net.)

A call has been made for a second edition of Edwards's classical treatise and the author, in this revised edition, has spared no effort to bring the work well into line with the latest and best thought. He has succeeded in setting forth clearly and briefly, those facts and principles that have been accepted by the medical profession as scientifically and clinically correct. There is a notable absence of theory, while there are no subjects discussed on which positive opinions have not been expressed, the aim of the author being to maintain a conservative rather than a radical attitude, in the discussion of questions not yet settled.

The methods of treatment are presented in such a way that they may be put into practice. We are now in an age of great therapeutical activity,—this has been appreciated and much care has been given the therapeutic section, which has been made sufficiently detailed and complete to give a useful representation of modern applied medicine. In the treatment of the various topics each has been given that amount of consideration to which its importance entitled it. There are many excellent textbooks on the practice of medicine and this one takes its place among the best. J. A. R.

